# Age-specific impact of COVID-19 on birth rates in Japan: An interrupted time-series analysis using national vital statistics

**DOI:** 10.1371/journal.pone.0341340

**Published:** 2026-01-21

**Authors:** Tasuku Okui

**Affiliations:** Medical Information Center, Kyushu University Hospital, Fukuoka City, Fukuoka Prefecture, Japan; Rikkyo University: Rikkyo Daigaku, JAPAN

## Abstract

**Objectives:**

A study investigating the impact of COVID-19 on birth rates across women’s age groups has not been previously conducted in Japan. Therefore, we examined this issue using national birth data.

**Methods:**

The analysis utilized monthly live birth data from the Vital Statistics from 2015 to 2023, which were accessed on July 27, 2025. We estimated the expected number of births in the post-pandemic period through quasi-Poisson regression analysis and calculated the ratios of the sum of actual births to that of predicted births for each women’s age group. In addition, a segmented regression analysis was performed to assess the effect of the pandemic on birth rates, focusing on changes in level and trend across age groups.

**Results:**

The ratios of the sum of actual births to that of predicted births in the post-pandemic period was close to one overall, but actual numbers were significantly lower than predicted numbers in women aged 15–19, 20–24, 25–29, and 35–39 years. Segmented regression analysis revealed significant declines in birth rates among women aged 15–19, 20–24, and 45–49 years due to the pandemic, with the rate ratios (RRs) being 0.880 (95% confidence interval (CI): 0.828, 0.935), 0.936 (95%CI: 0.907, 0.967), and 0.890 (95%CI: 0.824, 0.961), respectively, indicating that an immediate effect of the pandemic was evident in those age groups. In addition, significant trend changes were observed among women aged 20–24, 25–29, 35–39, and 45–49 years, with the RRs being 0.995 (95%CI: 0.993, 0.996), 0.998 (95%CI: 0.996, 0.999), 0.998 (95%CI: 0.997, 1.000), and 1.004 (95%CI: 1.001, 1.008), respectively, indicating an acceleration of the declining trend in some age groups.

**Conclusions:**

These findings suggest that the pandemic’s effect on birth rates varies by women’s age, with a more pronounced decrease among younger women.

## Introduction

The total fertility rates worldwide have shown a declining trend in recent decades [1], with birth rates decreasing particularly among high-income countries [2]. Japan has one of the lowest birth rate in the world [2]. The decline in birth rate is a major social issue in Japan, and it can lead to labor shortages and economic slowdown and jeopardize social security systems. Since the early 1970s, the number of births has been decreasing, with around 730,000 births in 2023—the lowest number on record [3]. It is important to analyze the reasons underlying Japan’s declining birth rate and implement appropriate measures.

The COVID-19 pandemic influenced birth rate trends, and studies investigating the effect of the pandemic have been conducted in many countries [4–10]. These studies generally reported declines in birth rates or total births due to the pandemic, though the magnitude of these effects varied among high-income countries [6]. Specifically, a decrease in crude birth rate during the pandemic period was not observed in countries such as the Netherlands and Finland [6]. A time-series analysis using data from major European countries showed that the number of births declined approximately nine months after the first wave of the pandemic [5]. In contrast, another study found that the pandemic’s impact on fertility intentions differed across European nations [11]. Specifically, the proportion of individuals who abandoned their fertility plans was particularly high, especially among those under 30 years of age in Italy, while a similar phenomenon was not observed in countries such as France and Germany [11].

Several studies have also been carried out in Japan to explore the impact of the COVID-19 pandemic on marriage and fertility [12–15]. A study examining birth trends after the pandemic found that actual number of births were significantly lower than the predicted numbers in January 2021 and May 2022 [12]. Another research investigating changes in the numbers of married, divorce and births from December 2011 to May 2021 indicated that the number of births significantly decreased from December 2020 to February 2021 [13], with regional differences in the extent of these changes. Additionally, a study investigating the trend in fertility rate after the COVID-19-related state of emergency showed that the fertility rate decreased during the first state of emergency, while it recovered in 2021 and 2022 [14]. Moreover, research indicates that the state of emergency declared due to the pandemic led to a 10.4% reduction in marriages per 1,000 people in Japan [15]. Furthermore, a study investigating the effect of the COVID-19 vaccine rollout on trends in fertility rate in Japan found no significant impact [16]. Conversely, a study investigating the effect of COVID-19 on birth rates across different women’s age groups has not been conducted in Japan. Studies examining the effects of COVID-19 on fertility by women’s age groups remain limited worldwide. However, a study in Finland found that fertility trends after the pandemic varied based on women’s age [17]. In addition, another study in Spain also showed that decline of births was concentrated on births such as first births and births to young and old mothers [4]. The social backgrounds of women—including school attendance, socioeconomic status, and use of infertility treatments—vary with age. Specifically, the pandemic’s impact may have been greater among younger women due to decreases in marriage rates, whereas older women may have been affected by the interruption of infertility treatments. Therefore, the effect of COVID-19 likely differed depending on women’s age, making it meaningful to examine trends in birth rates across different women’s age groups in Japan.

Thus, this study examined how the COVID-19 pandemic impacted birth rates trends across different women’s age groups in Japan using national birth data.

## Materials and methods

The analysis utilized live birth data from Japan’s Vital Statistics, covering the years 2015–2023 [18]. The data were accessed on July 27, 2025, and the author did not have access to any identifying information of individual participants during or after data collection. Data on live births reported to local governments in Japan are collected by the Ministry of Health, Labor and Welfare and published annually as Vital Statistics data. The analysis used the number of births categorized by maternal age group, birth month, and birth year. Data were available for the following age groups: less than 20 years, 20–24 years, 25–29 years, 30–34 years, 35–39 years, 40–44 years, and 45 years or older. The annual population of women across all 5-year age groups was obtained from the survey on population, demographics, and household numbers based on the Basic Resident Register [19]. Since the number of births among mothers under 15 and over 49 years of age was small, the birth counts for the < 20 and ≥45 years age groups were used to represent the 15–19 and 45–49 years categories. The number of births whose maternal age was uncertain was 18 in total in the periods.

To assess COVID-19’s impact on the pandemic, it was necessary to determine the onset time of the pandemic. Although the onset time varies among studies, April 2020 is frequently considered the onset time in Japan, coinciding with the declaration of the first state of emergency [20–22]. Therefore, April 2020 was considered as the start of the pandemic for this study. In contrast, a gestation generally takes 9 months, and the pandemic’s effect on birth rate was assumed to start 9 months after the pandemic’s onset in previous studies [6,16,23]. That is, if changes in human behaviors such as stopping or stepping childbirth occurred by the onset of the pandemic, it is assumed that their effects on birth rate appeared approximately 9 months after the onset of the pandemic. Therefore, January 2021 or later was defined as the post-pandemic period in this study, and December 2020 or earlier was defined as the pre-pandemic period in the analysis.

A retrospective time-series design using an interrupted time series analysis was employed as the study design. For the statistical analysis, we plotted monthly birth counts and birth rates by women’s age groups as well as for all ages combined. To compare actual birth rates with predicted values—derived from data before the pandemic onset—we used a quasi-Poisson regression model. A Poisson regression model is a regression model used for an outcome variable that is a count variable, and a quasi-Poisson regression model is an extended model of a Poisson regression model dealing with over-dispersion [24]. A quasi-Poisson regression model was used because over-dispersion was detected for many age groups in an over-dispersion test when using a Poisson regression model [25]. S1 Table shows the results of the over-dispersion test. In the model, the monthly number of births served as the outcome, with the month and time point as explanatory variables. The time point for January 2015 was set as one, and the value increased by one for each subsequent month. The logarithm of the women’s population was included as the offset term, and separate models were run for each women’s age group and for all ages combined. The logarithm of the women’s population was included as the offset term in the quasi-Poisson model to handle changes in population over time. By including the offset term, the association between birth rate and the explanatory variables can be modeled. We calculated the total actual births and the total predicted births after the pandemic began, and the ratio of sum of actual births to that of predicted births. We could assess the extent of the overall decrease or increase in births caused by the pandemic for each age group using the ratio. The 95% confidence interval (CI) of the ratio was derived by Monte Carlo simulation. Specifically, coefficients of the quasi-Poisson model were simulated by a multivariate normal distribution using the estimated coefficients and their variance-covariance matrix. The number of simulation was 1,000, and the seed was set as 1.

Additionally, a segmented regression analysis was performed as an interrupted time series analysis to assess the pandemic’s impact on birth rates [26]. This method can evaluate the level and trend changes of the outcome variable that result from the intervention. The number of births was considered the outcome, while time point, the indicator variable of the post-pandemic period, month, and an interaction term served as the explanatory variables. The coefficient of the intervention (the indicator variable of the post-pandemic period) reflects the change in birth rate level caused by the pandemic. The interaction term indicates the product of the indicator variable of the post-pandemic period and the number of months since January 2021, and its coefficient reflects the change in trend (slope) of the birth rate due to the pandemic. The logarithm of the women’s population served as the offset term, and a quasi-Poisson regression was performed separately for women’s age groups and all ages combined. The Ljung-Box test was conducted to assess the autocorrelation of the residuals derived from the quasi-Poisson model, and Newly-West standard errors were used to account for the autocorrelation [27,28]. Deviance residuals were calculated as the residuals, and the normality of the residuals was assessed by a Quantile-Quantile plot.

As a sensitivity analysis, we conducted a segmented regression analysis using Fourier terms (pairs of sine and cosine functions) as explanatory variables to account for seasonality instead of month [29,30]. In addition, as an additional analysis, we conducted a segmented regression analysis incorporating effects of the vaccine rollout. The time point of the vaccine rollout was set as June 2021 [31,32], and the effect was assumed to start 9 months later (March 2022). Therefore, the post-vaccination period was set as March 2022 or later. In addition to the explanatory variables used in the main segmented regression model, the model for the additional analysis included the indicator variable for the post-vaccination period and the interaction term between this indicator and the number of months since March 2022 as explanatory variables. The rate ratio (RR), 95% CI, and p-values were calculated for each explanatory variable. A p-value below 0.05 was considered statistically significant.

All analyses used R4.5.0 [33], with packages ggplot2, ggpubr, lmtest, performance, and sandwich [25,34–37]. Since publicly available data were utilized, no ethical approval from our institution was required [38].

During the preparation of this work, the author used ChatGPT in order to check grammar. After using this tool, the author reviewed and edited the content as needed.

## Results

**Fig 1:**
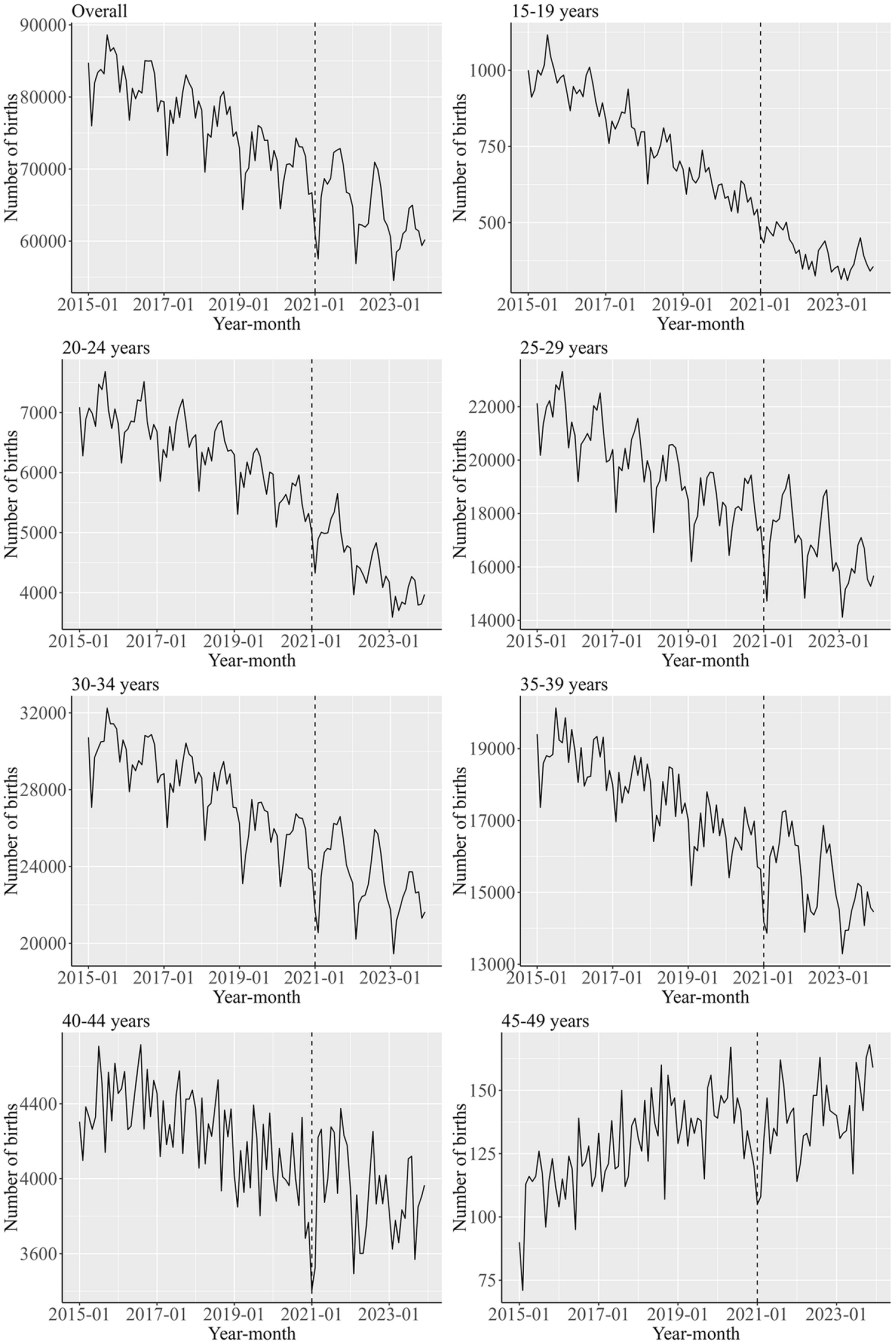
Trends in the number of births across different women’s age groups. The vertical dashed line indicates the cutoff point between the pre-pandemic and the post-pandemic periods. The overall indicates the results of women aged 15–49 years.

Fig 1 illustrates trends in the number of births across different women’s age groups. Women aged 30–34 years had the highest number of births. Overall, the number of births declined across most age groups, except for women aged 45–49 years. A notable drop in the number of births was observed around January 2021 in most groups, with the decline particularly evident among older women.

**Fig 2:**
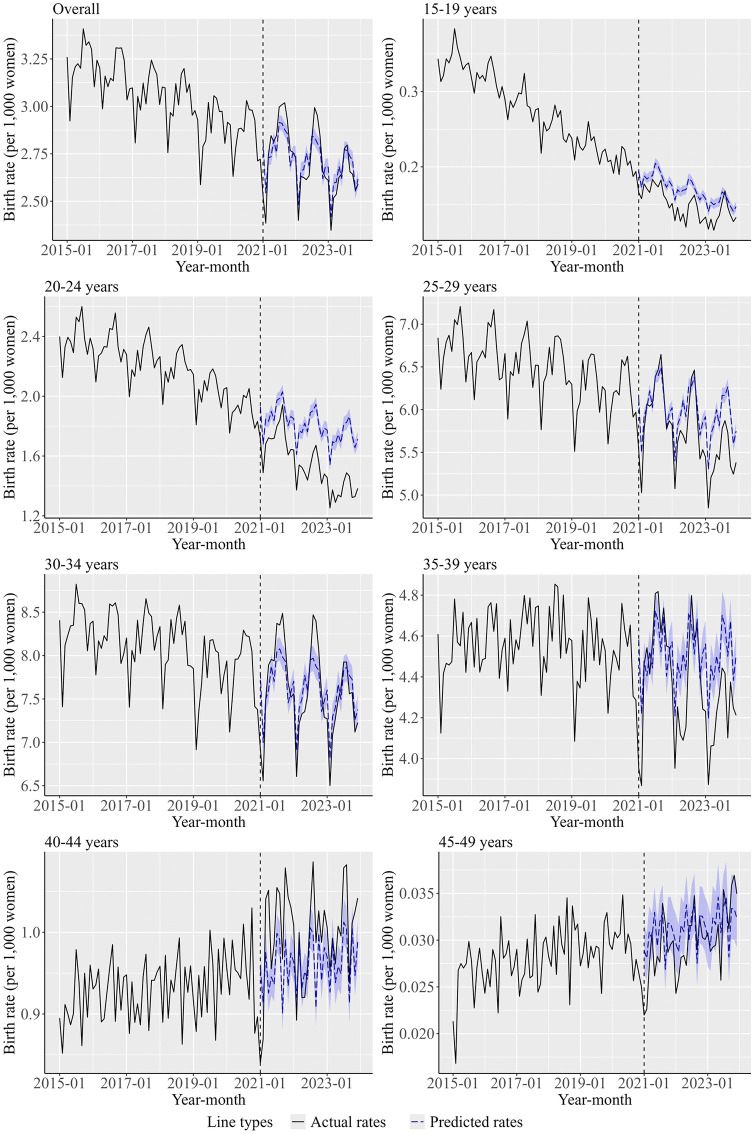
Trends in actual and predicted birth rates across different women’s age groups. The overall indicates the results of women aged 15–49 years. The birth rate represents the number of births per 1,000 women. The vertical dashed line marks the cutoff point between the pre-pandemic and the post-pandemic periods. In contrast, the blue dashed line shows the predicted birth rates derived from pre-pandemic data and shows the counterfactual values. The shaded areas indicate 95% CIs of the predicted values. A quasi-Poisson regression model was applied to the pre-pandemic data, and the predicted number of births was calculated based on the model. In the quasi-Poisson regression model, the month and time point were included as explanatory variables to take into account of time and seasonal effects, and the logarithm of the women’s population was included as the offset term.

Fig 2 illustrates the trends in actual and predicted birth rates across different women’s age groups, and S1 Fig illustrates the differences in actual and predicted number of births per 1,000 women in the post-pandemic period. The actual birth rate overall and among women aged 30–34 years was much lower than the predicted birth rate in January 2021, but it was higher than the predicted birth rate in September 2021 and 2022. For women aged 15–19, 20–24, 25–29, 35–39, and 45–49 years, the predicted number of births generally exceeded the actual numbers. Notably, the gap between the actual and predicted birth rates widened over the years for women aged 20–24 years. In addition, a notable decline in actual birth rates was observed in January 2021 among women aged 30 or older.

Table 1 present the sums of actual and predicted number of births in the post-pandemic period, along with the ratio of these two figures. For all ages combined, the ratio of actual to predicted births was 0.997 (95% CI: 0.987, 1.007), indicating a minimal difference between the two. Conversely, the ratios for women aged 15–19, 20–24, 25–29, and 35–39 years were 0.873 (95% CI: 0.851, 0.895), 0.852 (95% CI: 0.838, 0.867), 0.958 (95% CI: 0.949, 0.966), and 0.966 (95% CI: 0.952, 0.981), respectively, with the actual birth counts being lower than the predicted ones. These results indicate that approximately 13%, 15%, 4%, and 3% of the births were decreased by the pandemic among women aged 15–19, 20–24, 25–29, and 35–39 years, respectively.

**Table 1:** Sums of actual and predicted number of births in the post-pandemic period and their ratios.

Women’s age (years)	Sum of actual number of births	Sum of predicted number of births*	Ratio (95% CI)†
Overall	2,309,656	2,315,882	0.997 (0.987, 1.007)
15–19	14,452	16,550	0.873 (0.851, 0.895)
20–24	159,941	187,647	0.852 (0.838, 0.867)
25–29	602,276	628,730	0.958 (0.949, 0.966)
30–34	837,065	839,410	0.997 (0.985, 1.009)
35–39	550,027	569,425	0.966 (0.952, 0.981)
40–44	140,875	135,667	1.038 (1.022, 1.055)
45–49	5,020	5,261	0.954 (0.898, 1.012)

CI, confidence interval.

*A quasi-Poisson regression model using the month and time point as explanatory variables and women’s population as the offset term was applied to the pre-pandemic birth data, and the predicted number of births in the post-pandemic period was calculated.

†The ratio indicates the ratio of sum of actual number of births to sum of predicted number of births. The 95% CI of the ratio was derived by a Monte Carlo simulation. The simulation employed a multivariate normal distribution using the estimated coefficients of the quasi-Poisson regression model and their variance-covariance matrix.

Table 2 presents the findings of the segmented regression analysis. Notably, there was a statistically significant decrease in the birth rate among women aged 15–19, 20–24, and 45–49 years due to the pandemic, with the RRs being 0.880 (95% CI: 0.828, 0.935), 0.936 (95% CI: 0.907, 0.967), and 0.890 (95% CI: 0.824, 0.961), respectively. These results indicate that an immediate effect of the pandemic was particularly large among those age groups and indicate that the birth rate became 0.880, 0.936, and 0.890 times just after the pandemic among women aged 15–19, 20–24, and 45–49 years, respectively.

**Table 2:** Result of the segmented regression analysis.

	Pre-pandemic time effect (per month)		Level change due to the pandemic		Slope change of time effect due to the pandemic (per month)	
Women’s age (years)	RR (95% CI)	p-value	RR (95% CI)	p-value	RR (95% CI)	p-value
Overall	0.998 (0.998, 0.998)	<0.001	1.007 (0.975, 1.039)	0.685	1.000 (0.998, 1.001)	0.576
15–19	0.991 (0.991, 0.992)	<0.001	0.880 (0.828, 0.935)	<0.001	1.000 (0.996, 1.003)	0.820
20–24	0.996 (0.996, 0.997)	<0.001	0.936 (0.907, 0.967)	<0.001	0.995 (0.993, 0.996)	<0.001
25–29	0.998 (0.998, 0.999)	<0.001	0.996 (0.964, 1.029)	0.817	0.998 (0.996, 0.999)	0.004
30–34	0.999 (0.999, 0.999)	<0.001	1.008 (0.975, 1.042)	0.639	1.000 (0.998, 1.001)	0.522
35–39	1.000 (0.999, 1.000)	0.086	0.999 (0.962, 1.037)	0.947	0.998 (0.997, 1.000)	0.041
40–44	1.001 (1.000, 1.001)	0.010	1.034 (0.993, 1.076)	0.107	1.000 (0.999, 1.002)	0.618
45–49	1.002 (1.000, 1.003)	0.011	0.890 (0.824, 0.961)	0.003	1.004 (1.001, 1.008)	0.018

RR, rate ratio; CI, confidence interval.

The RR of the pre-pandemic time effect represents the monthly change in the birth rate in the pre-pandemic period. For example, for women aged 15–19 years, the RR was 0.991, indicating a 0.9% monthly decrease in the birth rate. The RR for the level change represents the immediate change in the birth rate due to the pandemic. For instance, the RR of 0.880 for women aged 15–19 years indicates that, after accounting for month and time effects, the birth rate in the post-pandemic period was 0.880 times that in the pre-pandemic period. In contrast, the RR for the slope change reflects an alteration in the trend of the birth rate following the pandemic. For women aged 15–19 years, the RR of 1.000 indicates that the post-pandemic time effect on birth rate was 1.000 times the pre-pandemic time effect, meaning the post-pandemic monthly RR was estimated as 0.991 × 1.000.

Additionally, statistically significant slope changes were observed among women aged 20–24, 25–29, 35–39, and 45–49 years due to the pandemic, with the RRs being 0.995 (95% CI: 0.993, 0.996), 0.998 (95% CI: 0.996, 0.999), 0.998 (95% CI: 0.997, 1.000), and 1.004 (95% CI: 1.001, 1.008), respectively. The results showed that the pandemic accelerated the declining trends in birth rates among women aged 20–24, 25–29, and 35–39 years, and the acceleration of the decrease in birth rate was the largest among women aged 20–24. The RR of 0.995 per month is equivalent to the RR of 0.94 per year, and it indicates the time effect on the birth rate was further reduced by 6% in a year by the pandemic.

S2 Table in the supplementary material indicates the results of the Ljung-Box tests derived from the residuals of the segmented regression analysis. There were autocorrelations in the residuals in many age groups, and it was suggested that it was appropriate to use the Newly-West standard errors to deal with the autocorrelation.

S2 Fig illustrates the Quantile-Quantile plots of the residuals derived from the segmented regression analysis by women’s age groups. The residuals tended to be normally distributed.

S3 Table in the supplementary material presents the results of the segmented regression analysis using Fourier terms. It was confirmed that the results were similar to that obtained by the model using month as an explanatory variable.

S4 Table in the supplementary material presents the findings of the segmented regression analysis incorporating the vaccine rollout effect. In contrast to the main segmented regression analysis not incorporating the vaccine rollout effect, the significant slope change of time effect by the pandemic was not observed among women aged 25–29, 35–39, and 45–49 years. In addition, there was a statistically significant decrease in birth rate due to the vaccine rollout among women aged 15–19 and 40–44 years. In addition, a statistically significant increasing slope change was observed among women aged 15–19 years, while a significant decreasing trend was observed among women aged 25–29 years.

## Discussion

We investigated the effect of the COVID-19 pandemic on birth rate trends in Japan. Our findings suggested that, overall, the COVID-19 pandemic did not significantly alter the general birth rate trend, but it did affect trends within specific age groups. We compare these results with previous studies and discuss potential reasons for these observations.

Japan is experiencing a declining birth rate over decades before the pandemic. The result of the interrupted time series analysis indicated that the pre-pandemic time effect was statistically significant for many age groups. Specifically, it indicated that a decreasing trend of the birth rate existed before the pandemic among women overall and those aged 15–19, 20–24, 25–29, and 30–34 years, while an increasing trend was observed in women aged 40–44 and 45–49 years. The result was consistent with the birth rate trend of each of the women’s age group in Japan. The interrupted time series analysis evaluated the level and slope changes after the pandemic taking into account of the pre-pandemic time effect.

The level change in birth rate was observed among women aged 15–19, 20–24, and 45–49 years, suggesting the immediate impact of the pandemic’s onset on birth rates among women of those ages. During COVID-19, social distancing, self-restraint, and staying at home were recommended nationwide [39–41]. Specifically, schools closed, and more people started remote work [42]. As a result, travel outside home decreased and duration of stay at home increased [43]. Our study revealed that the observed-to-predicted birth ratios were especially low for women aged 15–19 and 20–24 years during the pandemic. The result was contrary to that in California, which showed that birth counts showed an increasing trend compared with the expected values since 2021 among women aged < 25 years [44]. These age groups are strongly linked to premarital pregnancy in Japan [45], and there is a possibility that the pandemic has led to the decrease in interaction with others outside home and has affected the birth rate, particularly in these age groups. In addition, a previous study indicated that marriage rate in Japan decreased during this period [15]. Because the proportion of never-married women is higher at younger ages, it is considered that the decline in marriage due to the pandemic had a greater impact on the birth rate among younger women. Additionally, many countries, including Japan, have experienced a decline in fertility intentions during the pandemic [11,46–48]. In addition, a study in Japan found that the pandemic reduced birth planning among socially privileged households [48], and another study in Australia showed that parents with a child tended not to want to have another child due to the pandemic [46]. In addition, a study in Japan revealed that the employment rate of married women with children dropped more than that of those without children due to the pandemic [49], pointing out that childcare responsibilities further reduced the employment rate of mothers. In China, the decline in income caused by the pandemic became a factor preventing women from having children [50]. Therefore, the pandemic-related shifts in socioeconomic status could have influenced plans for having children.

Furthermore, the degree of level change in birth rate level was also large among women aged 45–49 years. The COVID-19 pandemic was associated with the decline in the number of births 9 months after the first-wave of the pandemic in European countries [5], and a significant drop in the number of births in January 2021 was also observed in Europe. A study in California also showed that a large decline in the number of births occurred in Winter 2020–2021 among women aged >=35 years [44]. One possible explanation is the decreased use of infertility treatments during this time. In Japan, the number of assisted reproductive procedures dropped in April and May 2020, which coincided with the initial declaration of state of emergency [51]. In addition, it was shown that the effect of the pandemic was larger in women aged 35 or older [51]. The percentage of women in Japan seeking medical assistance for fertility issues has been shown to be directly proportional to women’s age [52], and it is possible that the immediate effect of the pandemic was accentuated among women aged 45–49 years. A short-term drop in birth rate in December 2020 among women aged 35–39 was also observed in Spain, and it is pointed out that the closure of infertility clinics in March-May 2020 might be a cause [4].

The change in birth rate trends was observed among women aged 20–24, 25–29, 35–39, and 45–49 years, and it was suggested that the pandemic accelerated declines in women aged 20–24, 25–29, 35–39. Reduced opportunities for interaction with the opposite sex is a possible cause as mentioned previously. During the pandemic, government-issued priority measures to prevent the spread of the disease remained in effect until March 2022 [53], and the social movement of self-restraint continued until 2022. Additionally, the decline in fertility intention related to socioeconomic changes—a factor influencing level shifts—may also affect birth rate trends. Moreover, in many countries, social anxiety increased during COVID-19 [54], and that can affect marriage and childbirth. In contrast, the increasing trend was observed after the pandemic’s onset among women aged 45–49 years, and the reason is not certain. An increasing trend in birth rate was observed before the pandemic for those women due to the factors such as delayed marriage and childbearing and an increasing use of the infertility treatment, and the increasing trend may have contributed to the result.

Decreasing effects by the pandemic were not observed in women aged 30–34 years. A study using data from European countries also showed that the proportion of persons who were still planning to have a child after the onset of the pandemic in persons aged 30–34 was higher compared with that in younger persons [11]. One possible explanation is that women aged 30–34 are less affected by the decline in marriage caused by the pandemic compared to younger age groups. Additionally, they are less impacted by the closure of infertility clinics compared to women in older age groups. This age group had the highest number of births among all women’s age groups, their data had a large impact on the overall result of birth rate trends in Japan. In contrast, the number of births to women aged <25 years and those aged >=45 years was relatively small compared with that of the other age groups, and the changes in birth rates in women with those age groups did not have a large effect on the trend in birth rate for all ages combined.

The analysis incorporating the effects of vaccine rollout indicated that a decrease in birth rates was observed among women aged 15–19 and 40–44 years. It was suggested that the vaccine rollout did not contribute to the rise in birth rates, while the reason why it had a decreasing effect is not certain. It was discussed in a previous study that some symptoms caused by COVID-19 vaccines may have led to a reduced likelihood of conception [16]. Additionally, the analysis showed a change in the trend slope associated with the vaccine rollout among women aged 15–19 years, suggesting that the declining birth rates in this age group have slowed following the vaccine implementation.

Regarding changes in the medical service utilization by the pandemic, there were countries where the pandemic affected the contraceptive access [55,56], and an increase in the proportion of unintended pregnancy after the lockdown was reported in the United Kingdom [55]. In contrast, to the best of our knowledge, there is no evidence that the pandemic affected the contraceptive access in Japan. Condoms, which are available without medical service utilization, are by far the most prevalent method of contraceptives in Japan, and the prevalence of oral contraceptives or other modern contraceptives is low [57,58]. Therefore, it is considered that the effect of changes in contraceptive access by the pandemic on birth rate was smaller in Japan compared with that in other countries.

The analysis suggested that the overall birth rate was not affected by COVID-19, although the decline in birth rates accelerated in certain age groups in the post-pandemic period. This suggests that focusing on trends within specific age groups is more meaningful than examining the overall birth rate. It is also important to monitor whether the accelerated decline continues or stops in the future. Additionally, future studies should investigate trends in marriage rates across age groups. Moreover, conducting a similar analysis using data of other developed countries such as Italy and South Korea would be meaningful to understand the effect of the pandemic on birth rate.

A limitation of this study is that monthly birth data for mothers aged 15–19 and 45–49 years were not publicly available; thus, data for mothers under 20 years and over 44 years were used. The proportion of mothers aged <15 years among mothers under 20 years was 0.05% in the periods, and that of mothers aged >= 50 years among mothers aged >= 45 years was 3.65% [18]. Therefore, most births to mothers under 20 years and over 44 years were actually to mothers aged 15–19 and 45–49 years, respectively. Although the number of births to mothers aged 50 years or older is small, the increasing trend over the years may have partially contributed to the observed slope change in the time effect during the pandemic. Another limitation of the study is that it used aggregate time-series data, which may introduce ecological fallacy in the results. An ecological fallacy is a fallacy where a conclusion at the individual level is derived from an analysis at the group level [59], and the results in this study may not necessarily apply to individuals. Additionally, other factors such as administrative policies and economic trends in Japan could have influenced trends in birth rate. For example, infertility treatment began to be covered by insurance from April 2022, and women aged less than 43 years became the target of the policy [60]. In addition, a policy on childcare leave at birth for fathers was implemented in October 2022, and fathers are able to receive subsidies during the paternal leave by the policy [61]. It is possible that these policies have affected the birth rate. Furthermore, monthly birth data broken down by maternal age groups and regions were not publicly available, making regional analyses in future studies meaningful. Another limitation is that only yearly data were available for the women’s population, while the population changes monthly. Nevertheless, nationwide data from Japan were employed, providing insights into overall birth rate trends in Japan.

## Conclusion

This study examined the effects of the COVID-19 pandemic on birth rates trends across different women’s age groups in Japan using nationwide birth data. The ratio of sums of the actual and predicted number of births, calculated from pre-pandemic data, was lowest among women aged 20–24 years, implying that the pandemic effect was the largest in that age group. Additionally, segmented regression analysis revealed significant decreases in birth rates (level change) due to the pandemic among women aged 15–19, 20–24, and 45–49 years. Significant slope changes due to the pandemic were observed among women aged 20–24, 25–29, 35–39, and 45–49 years. These findings suggest that the pandemic’s impact on birth rates varied across age groups, with a tendency for a larger decrease among younger women. This underscores the importance of investigating the effects of the pandemic on birth rates by age groups, as well as exploring related factors such as marriage rates, fertility intentions, and economic influences in future studies. Additionally, monitoring whether these changes in birth rates during the pandemic will have long-term effects on fertility trends in Japan is also meaningful.

## Supporting information

S1 Table Results of the over-dispersion test. (PDF)

S2 Table Results of the Ljung-Box tests derived from the residuals of the segmented regression analysis. (PDF)

S3 Table Results of the segmented regression analysis using Fourier terms. (PDF)

S4 Table Results of the segmented regression analysis incorporating the vaccine rollout effect. (PDF)

S5 Table Dataset used in the analysis. (CSV)

S1 Fig Differences in actual and predicted number of births per 1,000 women in the post-pandemic period. (PDF)

S2 Fig Quantile-Quantile plots of the residuals derived from the segmented regression analysis by women’s age groups. (PDF)
