# Supplementary material for: Age-specific impact of COVID-19 on birth rates in Japan: An interrupted time-series analysis using national vital statistics
Source: PLoS One. 2026 Jan 21;21(1):e0341340. doi: 10.1371/journal.pone.0341340 (PMC12822959; doi:10.1371/journal.pone.0341340)
Supplement: S1 Table — (PDF) [file pone.0341340.s001.pdf]

S1 Table. Results of the over-dispersion test.

Women's age (years)	Dispersion ratio*	p-value
Overall	34.5	<0.001
15–19	1.4	0.004
20–24	4.1	<0.001
25–29	8.2	<0.001
30–34	16.9	<0.001
35–39	14.3	<0.001
40–44	4.6	<0.001
45–49	1.1	0.303

*Dispersion ratio over 1.0 indicates over-dispersion.
