# Supplementary material for: Age-specific impact of COVID-19 on birth rates in Japan: An interrupted time-series analysis using national vital statistics
Source: PLoS One. 2026 Jan 21;21(1):e0341340. doi: 10.1371/journal.pone.0341340 (PMC12822959; doi:10.1371/journal.pone.0341340)
Supplement: S2 Table — (PDF) [file pone.0341340.s002.pdf]

S2 Table. Results of the Ljung-Box tests derived from the residuals of the segmented regression analysis.

Women's age (years)	Chi-squared value	p-value
Overall	30.0	<0.001
15–19	4.0	0.046
20–24	35.3	<0.001
25–29	27.5	<0.001
30–34	36.0	<0.001
35–39	34.2	<0.001
40–44	14.9	<0.001
45–49	1.7	0.188
