# Supplementary material for: Age-specific impact of COVID-19 on birth rates in Japan: An interrupted time-series analysis using national vital statistics
Source: PLoS One. 2026 Jan 21;21(1):e0341340. doi: 10.1371/journal.pone.0341340 (PMC12822959; doi:10.1371/journal.pone.0341340)
Supplement: S3 Table — (PDF) [file pone.0341340.s003.pdf]

S3 Table. Results of the segmented regression analysis using Fourier terms.

Women's age (years)	Pre-pandemic time effect (per month)		Level change due to the pandemic		Slope change of time effect due to the pandemic (per month)	
	RR (95% CI)	p-value	RR (95% CI)	p-value	RR (95% CI)	p-value
Overall	0.998 (0.998, 0.998)	<0.001	1.006 (0.970, 1.044)	0.737	1.000 (0.998, 1.001)	0.606
15–19	0.991 (0.991, 0.992)	<0.001	0.882 (0.833, 0.934)	<0.001	1.000 (0.996, 1.003)	0.783
20–24	0.996 (0.996, 0.997)	<0.001	0.938 (0.907, 0.969)	<0.001	0.995 (0.993, 0.996)	<0.001
25–29	0.998 (0.998, 0.999)	<0.001	0.998 (0.964, 1.033)	0.888	0.998 (0.996, 0.999)	0.006
30–34	0.999 (0.998, 0.999)	<0.001	1.009 (0.975, 1.044)	0.619	1.000 (0.998, 1.001)	0.484
35–39	1.000 (0.999, 1.000)	0.157	0.998 (0.961, 1.037)	0.931	0.998 (0.997, 1.000)	0.030
40–44	1.001 (1.000, 1.001)	0.006	1.031 (0.989, 1.074)	0.152	1.001 (0.999, 1.002)	0.558
45–49	1.002 (1.001, 1.003)	0.008	0.880 (0.812, 0.954)	0.002	1.005 (1.001, 1.009)	0.024

RR, rate ratio; CI, confidence interval

The RR of the pre-pandemic time effect represents the monthly change in the birth rate in the pre-pandemic period. For example, for women aged 15–19 years, the RR was 0.991, indicating a 0.9% monthly decrease in the birth rate. The RR for the level change represents the immediate change in the birth rate due to the pandemic. For instance, the RR of 0.882 for women aged 15–19 years indicates that, after accounting for month and time effects, the birth rate in the post-pandemic period was 0.882 times that in the pre-pandemic period. In contrast, the RR for the slope change reflects an alteration in the trend of the birth rate following the pandemic. For women aged 15–19 years, the RR of 1.000 indicates that the post-pandemic time effect on birth rate was 1.000 times the pre-pandemic time effect, meaning the post-pandemic monthly RR was estimated as 0.991×1.000.
