# Supplementary material for: Age-specific impact of COVID-19 on birth rates in Japan: An interrupted time-series analysis using national vital statistics
Source: PLoS One. 2026 Jan 21;21(1):e0341340. doi: 10.1371/journal.pone.0341340 (PMC12822959; doi:10.1371/journal.pone.0341340)
Supplement: S4 Table — (PDF) [file pone.0341340.s004.pdf]

S4 Table. Results of the segmented regression analysis incorporating the vaccine rollout effect.

Women's age	Pre-pandemic time effect (per month)		Level change due to the pandemic		Slope change of time effect due to the pandemic (per month)	
	RR (95% CI)	p-value	RR (95% CI)	p-value	RR (95% CI)	p-value
Overall	0.998 (0.998, 0.998)	<0.001	0.983 (0.935, 1.034)	0.508	1.004 (0.997, 1.010)	0.274
15–19 years	0.991 (0.991, 0.992)	<0.001	0.926 (0.908, 0.945)	<0.001	0.994 (0.991, 0.997)	<0.001
20–24 years	0.996 (0.996, 0.997)	<0.001	0.922 (0.894, 0.952)	<0.001	0.998 (0.993, 1.002)	0.318
25–29 years	0.998 (0.998, 0.999)	<0.001	0.965 (0.924, 1.008)	0.112	1.003 (0.997, 1.009)	0.324
30–34 years	0.999 (0.999, 0.999)	<0.001	0.986 (0.929, 1.046)	0.645	1.003 (0.996, 1.011)	0.407
35–39 years	1.000 (1.000, 1.000)	0.044	0.970 (0.916, 1.027)	0.298	1.004 (0.997, 1.011)	0.318
40–44 years	1.001 (1.000, 1.001)	0.005	1.009 (0.956, 1.065)	0.739	1.005 (0.998, 1.013)	0.160
45–49 years	1.002 (1.000, 1.003)	0.010	0.880 (0.809, 0.957)	0.003	1.007 (0.994, 1.020)	0.275

Women’s age (years)	Level change due to the vaccine rollout		Slope change of time effect due to the vaccine rollout (per month)	
	RR (95% CI)	p-value	RR (95% CI)	p-value
Overall	0.965 (0.913, 1.019)	0.202	0.996 (0.989, 1.003)	0.236
15–19	0.931 (0.906, 0.956)	<0.001	1.013 (1.010, 1.016)	<0.001
20–24	0.957 (0.916, 0.999)	0.047	0.998 (0.994, 1.002)	0.317
25–29	0.971 (0.923, 1.021)	0.254	0.994 (0.988, 1.000)	0.046
30–34	0.971 (0.910, 1.037)	0.381	0.996 (0.988, 1.004)	0.334
35–39	0.948 (0.895, 1.003)	0.065	0.995 (0.988, 1.002)	0.179
40–44	0.936 (0.882, 0.994)	0.031	0.997 (0.989, 1.004)	0.362

45–49	0.952 (0.851, 1.066)	0.397	0.999 (0.986, 1.011)	0.830

RR, rate ratio; CI, confidence interval

The RR of the level change by the vaccine rollout indicates changes in the birth rate by the vaccine rollout. For example, for women aged 15–19 years, the RR was 0.931, indicating that the birth rate after the vaccine rollout was 0.931 times the birth rate from the post-pandemic period until the post-vaccination period. In contrast, the RR of the slope change of time effect by the vaccine rollout indicates changes in trends in the birth rate by the vaccine rollout. For women aged 15–19 years, the RR of 1.013 indicates that the time effect on birth rate after the vaccine rollout was 1.013 times the birth rate from the post-pandemic period until the post-vaccination period. Therefore, the RR of the post-vaccination time effect for women aged 15–19 years is estimated as $0.991 \times 0.994 \times 1.013$ per month.
