# Supplementary material for: Age-specific impact of COVID-19 on birth rates in Japan: An interrupted time-series analysis using national vital statistics
Source: PLoS One. 2026 Jan 21;21(1):e0341340. doi: 10.1371/journal.pone.0341340 (PMC12822959; doi:10.1371/journal.pone.0341340)
Supplement: S1 Fig — (PDF) [file pone.0341340.s006.pdf]

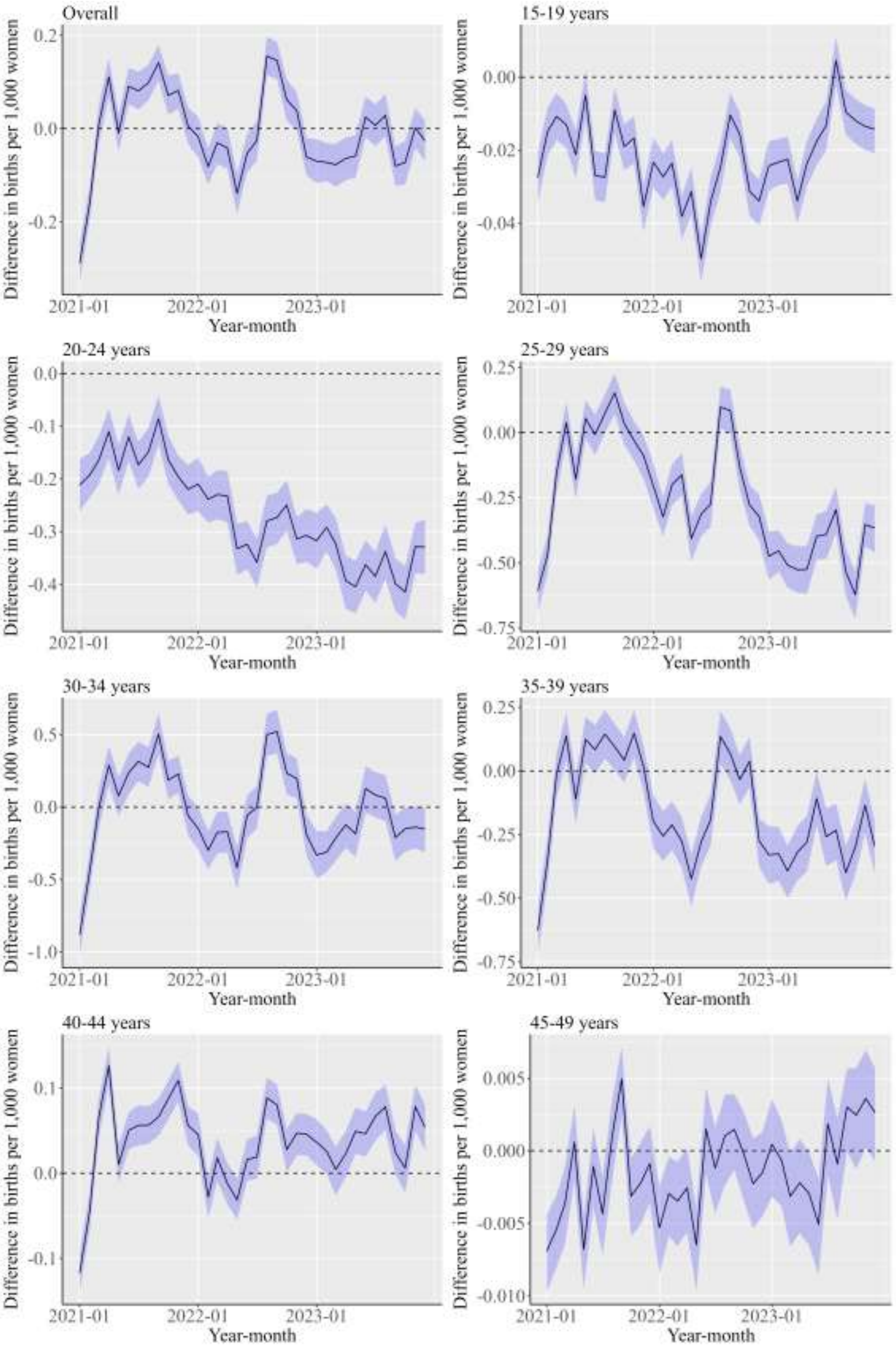

Overall
15-19 years
20-24 years
25-29 years
30-34 years
35-39 years
40-44 years
45-49 years
Difference in births per 1,000 women
Year-month
2021-01
2022-01
2023-01

S1 Fig. Differences in actual and predicted number of births per 1,000 women in the post-pandemic period.

The difference in births per 1,000 women indicates the difference in actual and predicted number of births per 1,000 women. The shaded areas indicate 95% CIs of the difference.
