# Supplementary material for: Age-specific impact of COVID-19 on birth rates in Japan: An interrupted time-series analysis using national vital statistics
Source: PLoS One. 2026 Jan 21;21(1):e0341340. doi: 10.1371/journal.pone.0341340 (PMC12822959; doi:10.1371/journal.pone.0341340)
Supplement: S2 Fig — (PDF) [file pone.0341340.s007.pdf]

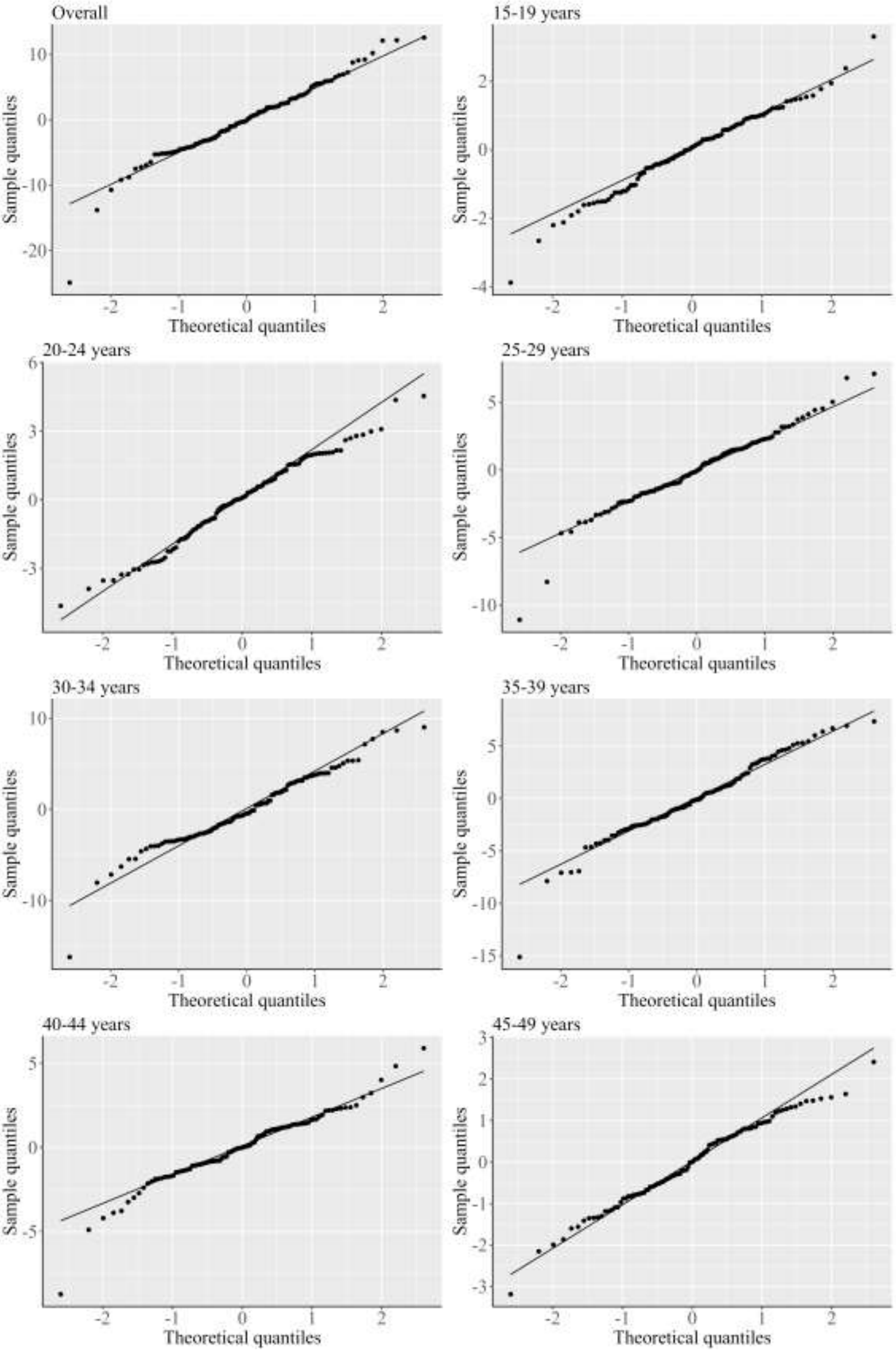
Overall
15-19 years
20-24 years
25-29 years
30-34 years
35-39 years
40-44 years
45-49 years
Sample quantiles
Theoretical quantiles

S2 Fig. Quantile-Quantile plots of the residuals of the segmented regression analysis by women's age groups.
